# The emergence of tomato flu: Another pandemic in making?

**DOI:** 10.1016/j.amsu.2022.104523

**Published:** 2022-08-31

**Authors:** Mrinmoy Kundu, Shankhaneel Ghosh, Soumyajit Das

**Affiliations:** Institute of Medical Sciences and SUM Hospital, Siksha ‘O’ Anusandhan, Bhubaneswar, India

## Correspondence

India has reported 82 cases of kids below five years of age and 26 children in the age group of 1–9 years with Tomato flu in Kerala and Bhubaneswar respectfully as of August 21st, 2022 [1]. This has made the entire globe aware that there may be a new outbreak in India. Instead of a viral illness, it can be a complication of dengue or chikungunya fever in children [2,3]. Another study has also reported that it could be a new variant of the viral hand, foot, and mouth disease [4].

As viral infections are widespread in children, they are most affected, however if it is not controlled and prevented, it might lead to negative effects on adults as well [5].Like other influenza strains, tomato flu is extremely infectious. The most likely method of its transmission is through close contact.

Its major symptoms, which include a high fever, rashes, and excruciating joint pain, are similar to those of chikungunya [2]. Tomato flu is named from the widespread appearance of painful, red blisters that eventually grow to the size of tomatoes. These blisters seem similar to those people get from the monkeypox virus [6]. Additional signs and symptoms are similar to those of other viral illnesses, such as tiredness, nausea, vomiting, diarrhoea, fever, dehydration, swelling of the joints, and body pains. These symptoms are also comparable to those of other viral diseases, such as dengue [3].

To stop the virus transmission, isolation must be used for five to seven days after the beginning of symptoms. The ideal method of prevention is maintaining good hygiene, sanitising the immediate area, and keeping the sick kid from exchanging objects, garments, food, or any other objects with other children who are not ill.

The most efficient and cost-efficient methods for safeguarding the general population against viral infections, particularly in children, the elderly, and those with impaired immune systems, are drug repurposing and vaccination. For the time being, there are no antiviral medications or vaccines available to treat or prevent tomato flu. Long term follow up is needed to better understand the morphology of the virus and potential treatment plans.

## Ethical approval

NA.

## Sources of funding

None.

## Author contribution

MK: designed the study. MK,SG,SD: made the first draft. MK and SG: updated the

Manuscript. MK, SG and SD: reviewed the final draft and edited final. All authors have critically reviewed and approved the final draft and are responsible for the content and similarity index of the manuscript.

## Consent

NA.

## Registration of research studies

1. Name of the registry: N/A
2. Unique Identifying number or registration ID: N/A
3. Hyperlink to your specific registration (must be publicly accessible and will bechecked): N/A

## Guarantor

NA.

## Provenance and peer review

Not commissioned, externally peer reviewed.

## Declaration of competing interest

None.
